# *N*-(2,3-Dichloro­phen­yl)-2,4-dimethyl­benzene­sulfonamide

**DOI:** 10.1107/S1600536810044338

**Published:** 2010-11-06

**Authors:** P. G. Nirmala, B. Thimme Gowda, Sabine Foro, Hartmut Fuess

**Affiliations:** a Department of Chemistry, Mangalore University, Mangalagangotri 574 199, Mangalore, India; b Institute of Materials Science, Darmstadt University of Technology, Petersenstrasse 23, D-64287 Darmstadt, Germany

## Abstract

In the title compound, C_14_H_13_Cl_2_NO_2_S, the dihedral angle between the two aromatic rings is 70.4 (1)°. The mol­ecular conformation is stabilized by an intra­molecular N—H⋯Cl hydrogen bond.

## Related literature

For the preparation of the compound, see: Savitha & Gowda (2006 ▶). For our study of the effect of substituents on the structures of *N*-(ar­yl)aryl­sulfonamides, see: Gowda *et al.* (2009 ▶); Nirmala *et al.* (2010**a* ▶,b* ▶). For related structures, see: Gelbrich *et al.* (2007 ▶); Perlovich *et al.* (2006 ▶).
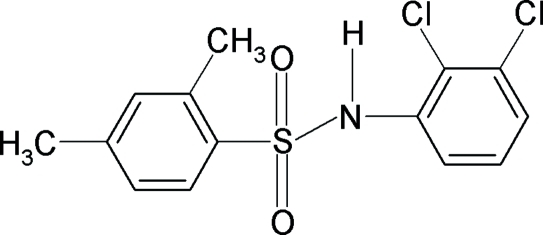


## Experimental

### 

#### Crystal data

C_14_H_13_Cl_2_NO_2_S
*M* *_r_* = 330.21
Monoclinic, 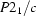

*a* = 9.198 (1) Å
*b* = 9.933 (1) Å
*c* = 16.099 (2) Å
β = 99.100 (1)°
*V* = 1452.4 (3) Å^3^
*Z* = 4
Cu *K*α radiation
μ = 5.37 mm^−1^
*T* = 299 K
0.53 × 0.20 × 0.20 mm

#### Data collection

Enraf–Nonius CAD-4 diffractometer
3394 measured reflections
2581 independent reflections
2367 reflections with *I* > 2σ(*I*)
*R*_int_ = 0.041
3 standard reflections every 120 min  intensity decay: 1.0%

#### Refinement

*R*[*F* ^2^ > 2σ(*F* ^2^)] = 0.042
*wR*(*F* ^2^) = 0.111
*S* = 1.13
2581 reflections
186 parameters
1 restraint
H atoms treated by a mixture of independent and constrained refinement
Δρ_max_ = 0.26 e Å^−3^
Δρ_min_ = −0.66 e Å^−3^

### 

Data collection: *CAD-4-PC* (Enraf–Nonius, 1996 ▶); cell refinement: *CAD-4-PC*; data reduction: *REDU4* (Stoe & Cie, 1987 ▶); program(s) used to solve structure: *SHELXS97* (Sheldrick, 2008 ▶); program(s) used to refine structure: *SHELXL97* (Sheldrick, 2008 ▶); molecular graphics: *PLATON* (Spek, 2009 ▶); software used to prepare material for publication: *SHELXL97*.

## Supplementary Material

Crystal structure: contains datablocks I, global. DOI: 10.1107/S1600536810044338/bt5400sup1.cif

Structure factors: contains datablocks I. DOI: 10.1107/S1600536810044338/bt5400Isup2.hkl

Additional supplementary materials:  crystallographic information; 3D view; checkCIF report

## Figures and Tables

**Table 1:** Hydrogen-bond geometry (Å, °)

*D*—H⋯*A*	*D*—H	H⋯*A*	*D*⋯*A*	*D*—H⋯*A*
N1—H1*N*⋯Cl1	0.84 (2)	2.50 (2)	2.9517 (19)	115 (2)

**Fig. 1.**
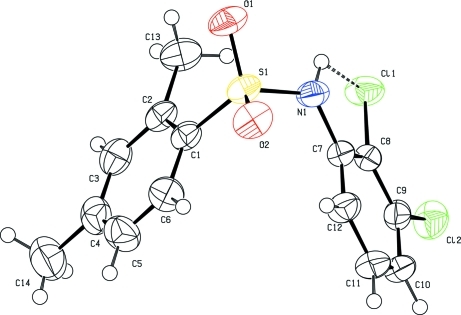
Molecular structure of the title compound, showing the atom labeling scheme. The displacement ellipsoids are drawn at the 50% probability level. The H atoms are represented as small spheres of arbitrary radii.

**Fig. 2.**
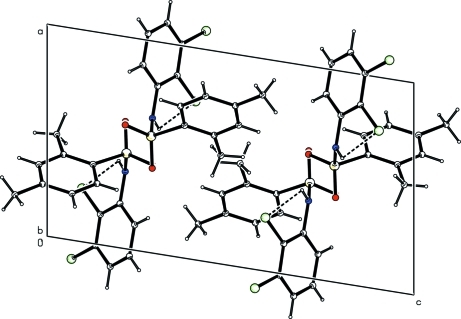
Molecular packing of the title compound with hydrogen bonding shown as dashed lines.

## supplementary crystallographic information

### Comment

As part of a study of the substituent effects on the structures of *N*-(aryl)arylsulfonamides (Gowda *et al.*, 2009; Nirmala *et al.*, 2010**a*,b*), in the present work, the structure of 2,4-dimethyl-*N*-(2,3-dichlorophenyl)benzenesulfonamide (I) has been determined (Fig. 1). The conformation of the N—C bond in the C—SO_2_—NH—C segment of the structure has *gauche* torsions with respect to the S═O bonds. The molecule is bent at the *N* atom with the C1—SO_2_—NH—C7 torsion angle of -50.3 (2)°, compared to the values of 46.1 (3)° (glide image of molecule 1) and 47.7 (3)° (molecule 2) in the two independent molecules of 2,4-dimethyl-*N*-(phenyl)benzenesulfonamide (II)(Gowda *et al.*, 2009), -54.9 (3)° in 2,4-dimethyl-*N*-(3,5-dichlorophenyl)benzenesulfonamide (III) (Nirmala *et al.*, 2010*b*), and 70.1 (2) and -66.0 (2)° in the two molecules of 2,4-dimethyl-*N*-(2,3-dimethylphenyl)- benzenesulfonamide (IV) (Nirmala *et al.*, 2010*a*)

The two benzene rings in (I) are tilted relative to each other by 70.4 (1)°, compared to the values of 67.5 (1)° (molecule 1) and 72.9 (1)° (molecule 2) in the two independent molecules of(II), 82.3 (1)° in (III), and 41.5 (1) and 43.8 (1)° in the two molecules of(IV). The other bond parameters in (I) are similar to those observed in (II), (III), (IV) and other aryl sulfonamides (Perlovich *et al.*, 2006; Gelbrich *et al.*, 2007). The crystal packing of molecules in (I) through N—H···O(S) hydrogen bonds (Table 1) is shown in Fig.2.

### Experimental

The solution of 1,3-xylene (1,3-dimethylbenzene) (10 ml) in chloroform(40 ml) was treated dropwise with chlorosulfonic acid (25 ml) at 0 ° C. After the initial evolution of hydrogen chloride subsided, the reaction mixture was brought to room temperature and poured into crushed ice in a beaker. The chloroform layer was separated, washed with cold water and allowed to evaporate slowly. The residual 2,4-dimethylbenzenesulfonylchloride was treated with 2,3-dichloroaniline in the stoichiometric ratio and boiled for ten minutes. The reaction mixture was then cooled to room temperature and added to ice cold water (100 ml). The resultant solid 2,4-dimethyl-*N*-(2,3-dichlorophenyl)benzenesulfonamide was filtered under suction and washed thoroughly with cold water. It was then recrystallized to constant melting point from dilute ethanol. The purity of the compound was checked and characterized by recording its infrared and NMR spectra (Savitha & Gowda, 2006).

The prism like colourless single crystals used in X-ray diffraction studies were grown in ethanolic solution by a slow evaporation at room temperature.

### Refinement

The H atom of the NH group was located in a difference map and its position refined with N—H = 0.86 (4) Å. The other H atoms were positioned with idealized geometry using a riding model with C—H = 0.93–0.96 Å All H atoms were refined with isotropic displacement parameters  set to 1.2 times of the *U*_eq_ of the parent atom.

### Crystal data

**Table:**

C_14_H_13_Cl_2_NO_2_S	*F*(000) = 680
*M**_r_* = 330.21	*D*_x_ = 1.510 Mg m^−^^3^
Monoclinic, *P*2_1_/*c*	Cu *K*α radiation, λ = 1.54180 Å
Hall symbol: -P 2ybc	Cell parameters from 25 reflections
*a* = 9.198 (1) Å	θ = 4.9–22.5°
*b* = 9.933 (1) Å	µ = 5.37 mm^−^^1^
*c* = 16.099 (2) Å	*T* = 299 K
β = 99.100 (1)°	Prism, colorless
*V* = 1452.4 (3) Å^3^	0.53 × 0.20 × 0.20 mm
*Z* = 4	

### Data collection

**Table:**

Enraf–Nonius CAD-4 diffractometer	*R*_int_ = 0.041
Radiation source: fine-focus sealed tube	θ_max_ = 66.9°, θ_min_ = 4.9°
graphite	*h* = −10→10
ω/2θ scans	*k* = −11→0
3394 measured reflections	*l* = −19→4
2581 independent reflections	3 standard reflections every 120 min
2367 reflections with *I* > 2σ(*I*)	intensity decay: 1.0%

### Refinement

**Table:**

Refinement on *F*^2^	Primary atom site location: structure-invariant direct methods
Least-squares matrix: full	Secondary atom site location: difference Fourier map
*R*[*F*^2^ > 2σ(*F*^2^)] = 0.042	Hydrogen site location: inferred from neighbouring sites
*wR*(*F*^2^) = 0.111	H atoms treated by a mixture of independent and constrained refinement
*S* = 1.13	*w* = 1/[σ^2^(*F*_o_^2^) + (0.0691*P*)^2^ + 0.3328*P*] where *P* = (*F*_o_^2^ + 2*F*_c_^2^)/3
2581 reflections	(Δ/σ)_max_ < 0.001
186 parameters	Δρ_max_ = 0.26 e Å^−^^3^
1 restraint	Δρ_min_ = −0.66 e Å^−^^3^

### Special details

**Table:**

Geometry. All e.s.d.'s (except the e.s.d. in the dihedral angle between two l.s. planes) are estimated using the full covariance matrix. The cell e.s.d.'s are taken into account individually in the estimation of e.s.d.'s in distances, angles and torsion angles; correlations between e.s.d.'s in cell parameters are only used when they are defined by crystal symmetry. An approximate (isotropic) treatment of cell e.s.d.'s is used for estimating e.s.d.'s involving l.s. planes.
Refinement. Refinement of *F*^2^ against ALL reflections. The weighted *R*-factor *wR* and goodness of fit *S* are based on *F*^2^, conventional *R*-factors *R* are based on *F*, with *F* set to zero for negative *F*^2^. The threshold expression of *F*^2^ > σ(*F*^2^) is used only for calculating *R*-factors(gt) *etc*. and is not relevant to the choice of reflections for refinement. *R*-factors based on *F*^2^ are statistically about twice as large as those based on *F*, and *R*- factors based on ALL data will be even larger.

### Fractional atomic coordinates and isotropic or equivalent isotropic displacement parameters (Å^2^)

**Table:**

	*x*	*y*	*z*	*U*_iso_*/*U*_eq_	
Cl1	0.25244 (6)	−0.10202 (6)	0.09534 (4)	0.05416 (19)	
Cl2	−0.08838 (6)	−0.14100 (6)	0.06647 (4)	0.0576 (2)	
S1	0.45032 (5)	0.26140 (5)	0.21630 (3)	0.04180 (18)	
O1	0.60015 (16)	0.22571 (19)	0.21346 (11)	0.0561 (4)	
O2	0.41640 (18)	0.33596 (18)	0.28705 (10)	0.0545 (4)	
N1	0.36407 (18)	0.11597 (18)	0.21294 (12)	0.0432 (4)	
H1N	0.409 (3)	0.052 (2)	0.1949 (16)	0.052*	
C1	0.3736 (2)	0.3504 (2)	0.12509 (13)	0.0385 (4)	
C2	0.3976 (2)	0.3127 (2)	0.04461 (13)	0.0418 (5)	
C3	0.3316 (3)	0.3909 (2)	−0.02241 (14)	0.0492 (5)	
H3	0.3482	0.3688	−0.0763	0.059*	
C4	0.2427 (3)	0.4999 (2)	−0.01297 (16)	0.0535 (6)	
C5	0.2205 (3)	0.5337 (2)	0.06777 (17)	0.0592 (6)	
H5	0.1606	0.6065	0.0757	0.071*	
C6	0.2859 (3)	0.4606 (2)	0.13590 (15)	0.0509 (5)	
H6	0.2713	0.4852	0.1897	0.061*	
C7	0.2086 (2)	0.10239 (19)	0.19856 (12)	0.0366 (4)	
C8	0.1436 (2)	0.00162 (19)	0.14533 (12)	0.0370 (4)	
C9	−0.0078 (2)	−0.0153 (2)	0.13289 (13)	0.0409 (5)	
C10	−0.0959 (2)	0.0673 (2)	0.17213 (14)	0.0482 (5)	
H10	−0.1975	0.0555	0.1634	0.058*	
C11	−0.0319 (2)	0.1676 (3)	0.22442 (15)	0.0513 (5)	
H11	−0.0910	0.2245	0.2506	0.062*	
C12	0.1197 (2)	0.1853 (2)	0.23870 (14)	0.0467 (5)	
H12	0.1617	0.2524	0.2751	0.056*	
C13	0.4877 (3)	0.1928 (3)	0.02611 (16)	0.0583 (6)	
H13A	0.5892	0.2075	0.0494	0.070*	
H13B	0.4789	0.1809	−0.0337	0.070*	
H13C	0.4526	0.1136	0.0508	0.070*	
C14	0.1715 (4)	0.5790 (3)	−0.0878 (2)	0.0778 (9)	
H14A	0.2434	0.5993	−0.1232	0.093*	
H14B	0.1324	0.6613	−0.0692	0.093*	
H14C	0.0933	0.5270	−0.1189	0.093*	

### Atomic displacement parameters (Å^2^)

**Table:**

	*U*^11^	*U*^22^	*U*^33^	*U*^12^	*U*^13^	*U*^23^
Cl1	0.0441 (3)	0.0501 (3)	0.0692 (4)	0.0080 (2)	0.0118 (3)	−0.0127 (2)
Cl2	0.0507 (3)	0.0612 (4)	0.0571 (3)	−0.0150 (2)	−0.0028 (2)	0.0009 (3)
S1	0.0302 (3)	0.0512 (3)	0.0432 (3)	−0.00367 (19)	0.00317 (19)	−0.0066 (2)
O1	0.0278 (7)	0.0756 (11)	0.0636 (10)	−0.0026 (7)	0.0036 (7)	−0.0020 (8)
O2	0.0526 (9)	0.0673 (10)	0.0427 (8)	−0.0068 (8)	0.0050 (7)	−0.0160 (7)
N1	0.0296 (8)	0.0444 (10)	0.0554 (10)	0.0026 (7)	0.0059 (7)	−0.0006 (8)
C1	0.0325 (9)	0.0398 (10)	0.0436 (10)	−0.0056 (8)	0.0072 (8)	−0.0065 (8)
C2	0.0340 (10)	0.0454 (11)	0.0474 (11)	−0.0083 (8)	0.0105 (8)	−0.0089 (9)
C3	0.0472 (12)	0.0552 (13)	0.0448 (11)	−0.0144 (10)	0.0067 (9)	−0.0055 (9)
C4	0.0542 (13)	0.0437 (12)	0.0602 (14)	−0.0143 (10)	0.0014 (11)	0.0032 (10)
C5	0.0653 (15)	0.0387 (11)	0.0729 (16)	0.0055 (11)	0.0092 (12)	−0.0036 (11)
C6	0.0575 (13)	0.0440 (11)	0.0527 (13)	0.0017 (10)	0.0136 (10)	−0.0096 (10)
C7	0.0292 (9)	0.0394 (10)	0.0415 (10)	0.0019 (7)	0.0070 (8)	0.0068 (8)
C8	0.0335 (10)	0.0376 (10)	0.0408 (10)	0.0050 (8)	0.0087 (8)	0.0084 (8)
C9	0.0359 (10)	0.0441 (10)	0.0419 (10)	−0.0037 (8)	0.0037 (8)	0.0107 (8)
C10	0.0295 (9)	0.0602 (13)	0.0564 (12)	0.0035 (9)	0.0110 (9)	0.0133 (10)
C11	0.0395 (11)	0.0573 (13)	0.0607 (13)	0.0094 (10)	0.0185 (10)	0.0021 (11)
C12	0.0402 (11)	0.0494 (12)	0.0528 (12)	0.0012 (9)	0.0143 (9)	−0.0042 (10)
C13	0.0533 (13)	0.0682 (15)	0.0563 (13)	0.0088 (12)	0.0179 (11)	−0.0144 (12)
C14	0.080 (2)	0.0654 (17)	0.082 (2)	−0.0069 (15)	−0.0054 (16)	0.0205 (15)

### Geometric parameters (Å, °)

**Table:**

Cl1—C8	1.7221 (19)	C5—H5	0.9300
Cl2—C9	1.733 (2)	C6—H6	0.9300
S1—O1	1.4307 (16)	C7—C12	1.389 (3)
S1—O2	1.4338 (16)	C7—C8	1.390 (3)
S1—N1	1.6448 (18)	C8—C9	1.385 (3)
S1—C1	1.763 (2)	C9—C10	1.375 (3)
N1—C7	1.419 (2)	C10—C11	1.375 (3)
N1—H1N	0.839 (17)	C10—H10	0.9300
C1—C6	1.387 (3)	C11—C12	1.387 (3)
C1—C2	1.399 (3)	C11—H11	0.9300
C2—C3	1.389 (3)	C12—H12	0.9300
C2—C13	1.507 (3)	C13—H13A	0.9600
C3—C4	1.379 (4)	C13—H13B	0.9600
C3—H3	0.9300	C13—H13C	0.9600
C4—C5	1.388 (4)	C14—H14A	0.9600
C4—C14	1.499 (4)	C14—H14B	0.9600
C5—C6	1.372 (4)	C14—H14C	0.9600
			
O1—S1—O2	119.02 (10)	C8—C7—N1	119.57 (17)
O1—S1—N1	104.09 (10)	C9—C8—C7	120.06 (18)
O2—S1—N1	108.37 (10)	C9—C8—Cl1	120.38 (16)
O1—S1—C1	111.01 (10)	C7—C8—Cl1	119.56 (15)
O2—S1—C1	107.10 (10)	C10—C9—C8	120.9 (2)
N1—S1—C1	106.58 (9)	C10—C9—Cl2	119.20 (16)
C7—N1—S1	123.88 (14)	C8—C9—Cl2	119.90 (17)
C7—N1—H1N	114.4 (18)	C9—C10—C11	119.14 (19)
S1—N1—H1N	114.7 (18)	C9—C10—H10	120.4
C6—C1—C2	120.5 (2)	C11—C10—H10	120.4
C6—C1—S1	117.09 (16)	C10—C11—C12	121.0 (2)
C2—C1—S1	122.40 (16)	C10—C11—H11	119.5
C3—C2—C1	117.1 (2)	C12—C11—H11	119.5
C3—C2—C13	118.4 (2)	C11—C12—C7	119.9 (2)
C1—C2—C13	124.5 (2)	C11—C12—H12	120.1
C4—C3—C2	123.2 (2)	C7—C12—H12	120.1
C4—C3—H3	118.4	C2—C13—H13A	109.5
C2—C3—H3	118.4	C2—C13—H13B	109.5
C3—C4—C5	118.1 (2)	H13A—C13—H13B	109.5
C3—C4—C14	120.9 (3)	C2—C13—H13C	109.5
C5—C4—C14	121.0 (3)	H13A—C13—H13C	109.5
C6—C5—C4	120.6 (2)	H13B—C13—H13C	109.5
C6—C5—H5	119.7	C4—C14—H14A	109.5
C4—C5—H5	119.7	C4—C14—H14B	109.5
C5—C6—C1	120.5 (2)	H14A—C14—H14B	109.5
C5—C6—H6	119.8	C4—C14—H14C	109.5
C1—C6—H6	119.8	H14A—C14—H14C	109.5
C12—C7—C8	119.07 (18)	H14B—C14—H14C	109.5
C12—C7—N1	121.33 (19)		
			
O1—S1—N1—C7	−167.74 (17)	C4—C5—C6—C1	−1.0 (4)
O2—S1—N1—C7	64.64 (19)	C2—C1—C6—C5	0.5 (3)
C1—S1—N1—C7	−50.33 (19)	S1—C1—C6—C5	−179.00 (19)
O1—S1—C1—C6	−138.08 (17)	S1—N1—C7—C12	−44.1 (3)
O2—S1—C1—C6	−6.64 (19)	S1—N1—C7—C8	137.91 (17)
N1—S1—C1—C6	109.19 (17)	C12—C7—C8—C9	0.0 (3)
O1—S1—C1—C2	42.39 (19)	N1—C7—C8—C9	177.98 (17)
O2—S1—C1—C2	173.83 (16)	C12—C7—C8—Cl1	−179.87 (16)
N1—S1—C1—C2	−70.35 (18)	N1—C7—C8—Cl1	−1.9 (2)
C6—C1—C2—C3	0.7 (3)	C7—C8—C9—C10	0.5 (3)
S1—C1—C2—C3	−179.79 (15)	Cl1—C8—C9—C10	−179.67 (16)
C6—C1—C2—C13	−178.4 (2)	C7—C8—C9—Cl2	179.99 (14)
S1—C1—C2—C13	1.1 (3)	Cl1—C8—C9—Cl2	−0.2 (2)
C1—C2—C3—C4	−1.5 (3)	C8—C9—C10—C11	−0.1 (3)
C13—C2—C3—C4	177.6 (2)	Cl2—C9—C10—C11	−179.59 (17)
C2—C3—C4—C5	1.0 (3)	C9—C10—C11—C12	−0.8 (3)
C2—C3—C4—C14	−178.6 (2)	C10—C11—C12—C7	1.2 (3)
C3—C4—C5—C6	0.3 (4)	C8—C7—C12—C11	−0.8 (3)
C14—C4—C5—C6	179.9 (3)	N1—C7—C12—C11	−178.8 (2)

### Hydrogen-bond geometry (Å, °)

**Table:**

*D*—H···*A*	*D*—H	H···*A*	*D*···*A*	*D*—H···*A*
N1—H1N···Cl1	0.84 (2)	2.50 (2)	2.9517 (19)	115 (2)
